# Microbiota and metabolites alterations in proximal and distal gastric cancer patients

**DOI:** 10.1186/s12967-022-03650-x

**Published:** 2022-09-30

**Authors:** Yan Yang, Daofeng Dai, Wen Jin, Yingying Huang, Yingzi Zhang, Yiran Chen, Wankun Wang, Wu Lin, Xiangliu Chen, Jing Zhang, Haohao Wang, Haibin Zhang, Lisong Teng

**Affiliations:** 1 Department of Surgical Oncology, The First Affiliated Hospital, School of Medicine, Zhejiang University, Hangzhou, 310003 China; 2 Clinical Research Center, The First Affiliated Hospital, School of Medicine, Zhejiang University, Hangzhou, 310003 China; 3 Jiangxi Otorhinolaryngology Head and Neck Surgery Institute, Department of Otorhinolaryngology-Head and Neck Surgery, The First Affiliated Hospital of Nanchang University, Nanchang, Jiangxi China; 4 The First Affiliated Hospital, School of Medicine, Zhejiang University, Hangzhou, 310003 China

**Keywords:** Proximal gastric cancer, Distal gastric cancer, Microbiome, Metabolomics

## Abstract

**Background:**

Globally, gastric cancer is the third most common cancer and the third leading cause of cancer death. Proximal and distal gastric cancers have distinct clinical and biological behaviors. The microbial composition and metabolic differences in proximal and distal gastric cancers have not been fully studied and discussed.

**Methods:**

In this study, the gastric microbiome of 13 proximal gastric cancer tissues, 16 distal gastric cancer tissues, and their matched non-tumor tissues were characterized using 16S rRNA amplicon sequencing. Additionally, 10 proximal gastric cancer tissues, 11 distal gastric cancer tissues, and their matched non-tumor tissues were assessed by untargeted metabolomics.

**Results:**

There was no significant difference in microbial diversity and richness between the proximal and distal gastric cancer tissues. At the genus level, the abundance of *Rikenellaceae_RC9_gut_group*, *Porphyromonas*, *Catonella*, *Proteus*, *Oribacterium,* and *Moraxella* were significantly increased in Proximal T, whereas that of *Methylobacterium_Methylorubrum* was significantly increased in Distal T. The untargeted metabolomics analysis revealed 30 discriminative metabolites between Distal T and Distal N. In contrast, there were only 4 discriminative metabolites between Proximal T and Proximal N. In distal gastric cancer, different metabolites were scattered through multiple pathway, including the sphingolipid signaling pathway, arginine biosynthesis, protein digestion and absorption, alanine, aspartate and, glutamate metabolism, etc.In proximal gastric cancer, differential microbial metabolites were mainly related to hormone metabolism.

**Conclusion:**

*Methylobacterium-Methylorubrum* was significantly increased in Distal T, positively correlated with cancer-promoting metabolites, and negatively correlated with cancer-inhibiting metabolites. *Rikenellaceae_RC_gut_group* was significantly increased in Proximal T and positively correlated with cancer-promoting metabolites. Further studies regarding the functions of the above-mentioned microorganisms and metabolites were warranted as the results may reveal the different mechanisms underlying the occurrence and development of proximal and distal gastric cancers and provide a basis for future treatments.

**Importance:**

First, the differences in microbial composition and metabolites between the proximal and distal gastric cancers were described; then, the correlation between microbiota and metabolites was preliminarily discussed. These microbes and metabolites deserve further investigations as they may reveal the different mechanisms involved in the occurrence and development of proximal and distal gastric cancers and provide a basis for future treatments.

**Supplementary Information:**

The online version contains supplementary material available at 10.1186/s12967-022-03650-x.

## Introduction

Gastric cancer (GC) is the third most common cancer globally and the third leading cause of cancer death [1]. China is a high-incidence area of GC [2]. GC can be divided into proximal GC, middle GC, and distal GC [3]. It is generally believed that proximal GC exhibits different clinical and biological behaviors compared with middle and distal GC. A study in Portugal indicated that proximal and distal gastric cancers are significantly different in terms of patient survival, tumor size, venous invasion, nodal status, and overall stage [4]. Moreover, the prognosis for proximal GC is significantly worse than distal GC [3].

Previous studies have also investigated the molecular differences between proximal and distal GCs. Whole-genome sequencing analysis of gastroesophageal junction (GEJ) carcinoma and distal GC by previous researchers showed no significant difference between the mutations in the two cancers and that the expression rates of PD-L1 in distal GC and GEJ cancer were 58.3% and 66.7%, respectively [5]. Moreover, the expression of the adenomatous polyposis coli gene, β-catenin, and E-cadherin was not significantly different between proximal and distal GCs [6]. An analysis conducted by Zhao et al. based on the SEER and TCGA databases revealed that the prognosis of proximal GC was worse than distal GC. Among the 280 differential genes, 90 were up-regulated in distal GC, while 190 were up-regulated in proximal GC. Pathway analysis showed that the activity of serine protease, ion channel (Na + /Cl-), and cytoskeleton could be related to the poor prognosis of proximal GC [7]. Furthermore, lower blood glucose levels were significantly associated with an increased risk of distal GC [8]. The overexpression of HER2 was significantly higher in Chinese patients with proximal GC than those with distal GC [9].

GC is a multifactorial disease, and alterations in the tumor microenvironment are necessitated for GC initiation, progression, and metastasis [10, 11]. As part of the tumor microenvironment, gastric microbiota has attracted increasing attention as it can impact cancer growth and spread in several ways. However, gastric microbiota has been relatively understudied compared to gut microbiota. Although the human stomach is thought to be exclusively inhabited by *Helicobacter pylori* (Hp) and viewed as an inhospitable environment for microbiota, attributable to its acidic conditions and other antimicrobial factors, more gastric microbiota has been identified with the development of sequencing technology; studies have demonstrated that gastric bacteria mainly include *Proteus, Firmicutes, Bacteroidetes, Actinomycetes,* and *Clostridium *[12]. Currently, it is unclear whether there is a correlation between the diversity of the gastric microbiota and the progression from healthy gastric mucosa to gastric cancer. Some studies found GC microbiota exhibit decreased microbial diversity, decreased abundance of Hp, and enrichment with other bacteria [13, 14]. However, there have also been studies suggesting that gastric cancer is associated with increased diversity and richness of the microbiota [15]. There were also research results indicated there was no significant difference in microbial abundance between gastric cancer and control samples [16].

The metabolome of GC has been studied as well; Pan et al. discovered that TG (54:2), G3p, α- aminobutyric acid, α-CEHC, dodecanol, glutamylalanine, 3-methylalanine, sulfite, CL (63:4), PE NME (40:5), TG (53:4), retinol, 3-hydroxysterol, tetradecanoic acid, Mg (21:0 / 0:0 / 0:0), tridecanoic acid, myristic acid glycine, and octacarbonate were potential biomarkers of abdominal metastasis from GC[17]. In another instance, Lee et al. studied the correlation between bile acid metabolism and GC [18]. A study comparing urine metabolomics of patients with GC and healthy controls found significant differences in urine alanine, citric acid, creatine, creatinine, glycerol, hippuric acid, phenylalanine, taurine, and 3-hydroxybutyric acid between the two groups [19]. In general, metabolic changes in blood, urine, gastric juice, and tissue in patients with GC have been evaluated, and changes in the metabolic spectrum have also been validated to be related to the occurrence and development of GC [20].

The prognostic differences and molecular biological characteristics of proximal and distal GCs have been explored for decades; the microbiome and metabolome of GC have also been studied [21]. However, the microbial composition and metabolic differences between proximal and distal GCs have not been fully studied and discussed. This study aimed to explore the metabolic differences between microbial-related proximal and distal gastric cancer through 16S rRNA amplicon sequencing and non-targeted metabolome analysis and explore the causes and development of proximal and distal gastric cancer.

## Methods

### Samples

16 distal gastric cancer (GC) patients and 13 proximal GC patients with no history of preoperative chemotherapy were enrolled from January 2018 to August 2019 at the First Affiliated Hospital, School of Medicine, Zhejiang University. All GC patients were diagnosed by postoperative pathological examinations. The clinical-pathological features of GC, such as tumor stage, tumor differentiation, and Lauren classification of tumor type, were collected. Baseline characteristics, including age, gender, body mass index (BMI), and history of hypertension and diabetes, were recorded as well. The clinical and pathological staging was in accordance with the 8^th^ edition of the American Joint Committee on Cancer (AJCC) TNM staging system for gastric cancer. The exclusion criteria were as follows: BMI > 30; use of antibiotics, probiotics, prebiotics, or synbiotics in the previous month; preoperative chemotherapy, radiotherapy, or other biological treatment prior to gastrectomy. Basic demographic and clinical data were collected at the time of inclusion (Additional file 1: Table S1). The research was approved by the Ethics Committee of the First Affiliated Hospital, School of Medicine, Zhejiang University (IIT20200503A). Informed written consent was obtained from each patient before enrollment.

### DNA extraction, 16S detection, and data analysis

After thoroughly grinding and breaking 25 mg of tissue, total genomic DNA was extracted using a DNA Extraction Kit according to the manufacturer’s instructions (Qiagen). DNA concentration was verified with NanoDrop and agarose gel. The genomic DNA was used as the template for PCR amplification with the barcoded primers and DNA Polymerase (Takara). For bacterial diversity analysis, the V3-V4 variable regions of 16S rRNA genes were amplified with universal primers 343F and 798R. Finally, the library was sequenced on an Ion S5TM XL platform, and 400–600 bp single-end reads were generated.

Raw sequencing data were in the FASTQ format. Paired-end reads were then preprocessed using Cutadapt to detect and cut off ambiguous bases (N); low-quality sequences with an average quality score below 20 were also cut off. The chimeric sequence was subsequently removed from reads obtained after the above processing. The reads sequence was then compared with the species annotation database to detect the chimera sequence, which was finally removed to obtain the final effective data (clean reads). QIIME software (version 1.8.0) was utilized to investigate species diversity and evaluate differences in microbial community composition. Bioinformatics analysis was performed with the OECloud tools at https://cloud.oebiotech.cn.

### Metabolome detection and data analysis

100 mg of tissue samples were used to extract the metabolites. The extracted samples were stored at -80 ℃ for further use. UHPLC-MS/MS analysis was performed using a Vanquish UHPLC system coupled with an Orbitrap Q Exactive series mass spectrometer (Thermo Fisher). The samples were injected into a Hyperil Gold column at a flow rate of 0.2 mL/min. The eluents for the positive polarity mode were eluent A (0.1% FA in water) and eluent B (methanol). The eluents for the negative polarity mode were eluent A (5 mM ammonium acetate, pH 9.0) and eluent B (methanol). The solvent gradient was set as follows: 2% B, 1.5 min; 2% to 100% B, 12.0 min; 100% B, 14.0 min; 2% to 100% B, 14.1 min; 2% B, 17 min. The Q Exactive mass spectrometer operated in both positive and negative polarity modes; the spray voltage was set to 3.2 kV, and the capillary temperature was set to 320 °C. Compound Discoverer 3.1 (Thermo Fisher) was used to process the original data file generated by UHPLC-MS/MS and perform peak calibration, peak picking, and quantification for each metabolite. Next, the peak intensity was normalized to the total spectral intensity. The molecular formula was predicted using the normalized data based on additive ions, molecular ion peaks, and fragment ions. Thereafter, peaks were matched using mzCloud, mzVault, and MassList databases to acquire accurately qualitative and relatively quantitative results. These metabolites were annotated using the Kyoto Encyclopedia of Genes and Genomes (KEGG) database, the Human Metabolome Database (HMDB), and the the LIPID MAPS Structure Database (LMSD). Orthogonal Partial Least Squares-Discriminant Analysis (OPLS-DA) was performed. The metabolites with variable importance in projection (VIP) > 1 and p-value < 0.05 and fold change ≥ 2 or FC ≤ 0.5 were considered differential metabolites. The functions of these metabolites and metabolic pathways were analyzed using the KEGG database.

### Combined microbiome-metabolome analysis

The correlation between the different microbiota and metabolites was calculated according to the one-to-one correspondence relationship between samples. Spearman correlation calculation method was utilized for the correlation algorithm.

## Results

### Differences in microbial diversity, richness, and composition between proximal and distal gastric cancers

**Fig. 1:**
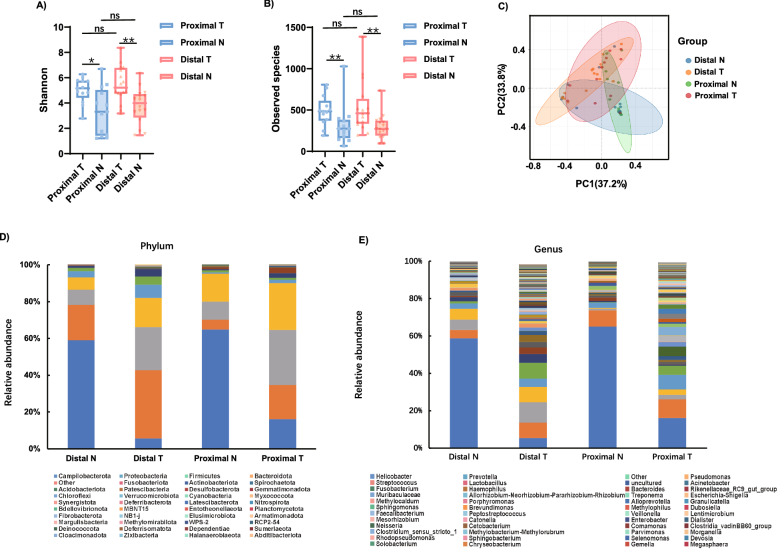
Altered gastric microbiota in 16 distal gastric cancer tissues, 13 proximal gastric cancer tissues compared with matched non-tumor tissues. **A**, **B** The observed species and Shannon indices were used to evaluate the microbial diversity of the proximal gastric cancer tissues, distal gastric cancer tissues and matched non-tumor tissues. **C** PCoA of weighted UniFrac distance demonstrated that the proximal, distal tumor tissues and matched non-tumor tissues showed four distinct clusters. **D**, **E** The microbial relative abundance of proximal, distal tumor tissues and matched non-tumor tissues at the phylum and genus levels. Proximal T,proximal GC tumor tissues; Proximal N,proximal GC non-tumor tissues; Distal T, distal GC tumor tissues; Distal N, distal GC non-tumor tissues

Alpha diversity was analyzed to investigate the differences in microbial diversity between the groups. The Shannon index, which reflects species richness and evenness, was higher in the distal GC tissues (Distal T) than in the matched non-tumor tissues (Distal N) (p = 0.0014). Shannon index was higher in the proximal GC tissues (Proximal T) than in the matched non-tumor tissues (Proximal N) (p = 0.0164). However, the Shannon index of Proximal T was not significantly different compared with Distal T (p = 0.5306) (Fig. 1A). Similarly, the Shannon index of Distal N was not significantly different compared with Proximal N (p = 0.4811)(Fig. 1A).The observed species, which reflected the species richness, was not significantly different between distal GC and proximal GC (Distal T vs Proximal T, p = 0.6703;Distal N vs Proximal N,p = 0.8462); but the observed species in GC tumor tissues was significantly different from the matched non-tumor tissues (Distal T vs. Distal N, p = 0.0039; Proximal T vs. Proximal N, p = 0.0072) (Fig. 1B). In order to compare the composition of the microbial community between the proximal and distal GC tissues, beta diversity analysis was performed. Significant clustering was detected for the Weighted_unifrac PCoA analysis between Proximal T, Distal T, and the matched non-tumor tissues (Fig. 1C). The taxonomic profiles of the gastric microbiota are illustrated in Figs. 1D, E. At the phylum level, the top 5 gastric microorganisms identified in Distal N and Distal T were *Campilobacterota*, *Proteobacteria*, *Firmicutes*, *Bacteroidota,* and *Others*. The top 5 gastric microorganisms in Proximal N and Proximal T were *Campilobacterota*, *Bacteroidota*, *Firmicutes*, *Proteobacteria,* and *Spirochaetota* (Fig. 1D). At the genus level, the 10 most prevalent microorganisms in Distal N were *Helicobacter*, *Pseudomonas*, *Other*, *Prevotella*, *Streptococcus*, uncultured, *Escherichia-Shigella*, *Fusobacterium*, *Allorhizobium-Neorhizobium-Pararhizobium-*

*Rhizobium,* and *Lactobacillus*. The 10 most common microorganisms in Distal T were *Other*, *Lactobacillus*, *Pseudomonas*, *Prevotella*, *Helicobacter*, uncultured, *Streptococcus*, *Haemophilus*, *Acinetobacter*, and *Fusobacterium*. *Helicobacter*, *Prevotella*, *Streptococcus*, *Porphyromonas*, *Acinetobacter*, *Alloprevotella*, *Lentimicrobium*, *Treponema*, *Peptostreptococcus,* and *Fusobacterium* were the top 10 microorganisms detected in Proximal N. Lastly, the 10 most common microorganisms in Proximal T were *Helicobacter*, *Prevotella*, *Streptococcus*, *Rikenellaceae_RC9_gut_group*, *Lactobacillus*, *Methylocaldum*, *Treponema*, *Pseudomonas*, *Methylophilus,* and *Sphingomonas* (Fig. 1E). We provided stacked bar plots for all samples in phylum level and genus level (Additional files2: Fig. S1 and Additional files 3: Fig.S2).

### Analysis of the differential taxa between distal T and distal N

**Fig. 2:**
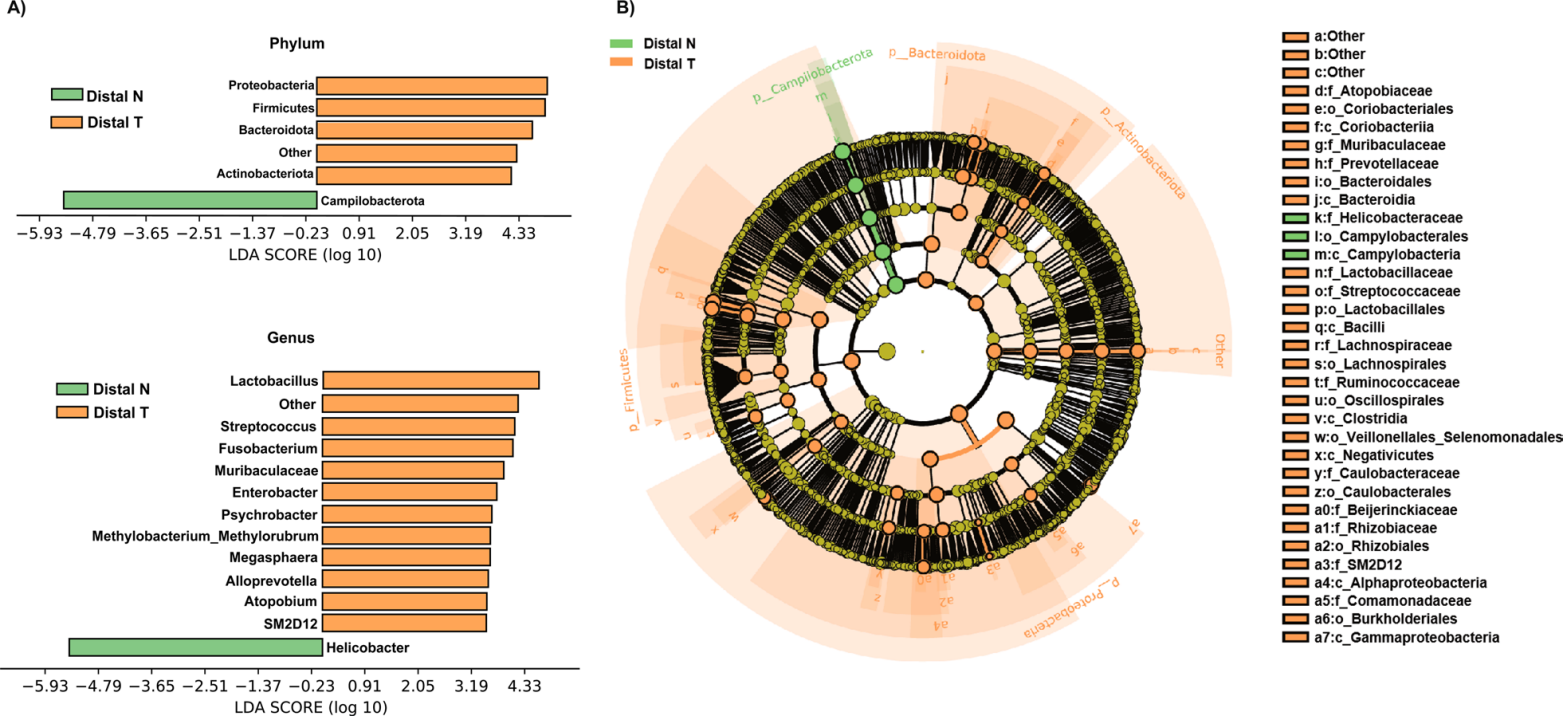
Differential microbiota of Distal T and Distal N. **A**, **B** Differential taxa at genus and phylum levels and cladogram identified by LEfSe analysis (LDA > 3.0, Q < 0.05)

The linear discriminant analysis (LDA) effect size (LEfSe) method (LDA > 3.0, corrected p-value < 0.05) was used to analyze the composition of the flora in Distal T, and Distal N. At the phylum level, the abundance of *Firmicutes*, *Bacteroidota*, *Proteobacteria*, *Other*, and *Actinobacteriota* was significantly increased in Distal T, whereas only the abundance of *Campilobacterota* was significantly decreased in Distal T. At the genus level, the abundance of *Lactobacillus, Other, Streptococcus, Fusobacterium, Muribaculaceae, Enterobacter, Psychrobacter, Methylobacterium_Methylorubrum, Megasphaera, Alloprevotella, Atopobium,* and *SM2D12* was significantly increased in Distal T, while the abundance of *Helicobacter* was significantly decreased in Distal T (Fig. 2A, B and Additional file 4: Figure S3A).

### Analysis of the differential taxa between proximal T and proximal N

**Fig. 3:**
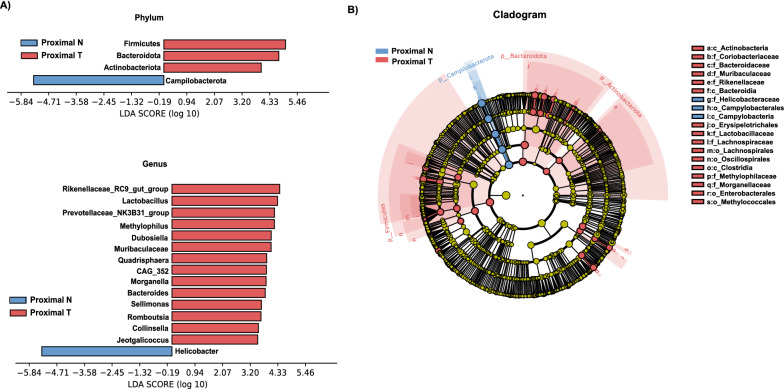
Differential microbiota of Proximal T and Proximal N. **A**, **B** Differential taxa at genus and phylum levels and cladogram identified by LEfSe analysis (LDA > 3.0, Q < 0.05)

Similarly, the linear discriminant analysis (LDA) effect size (LEfSe) method (LDA > 3.0, corrected p-value < 0.05) was used to analyze the composition of the flora in Proximal T and Proximal N. At the phylum level, the abundance of *Firmicutes, Bacteroidota,* and *Actinobacteriota* were significantly increased in Proximal T, while that of *Campilobacterota* was significantly decreased in Proximal T. At the genus level, the abundance of *Rikenellaceae_RC9_gut_group*, *Lactobacillus*, *Methylophilus*, *Dubosiella*, *Prevotellaceae_NK3B31_group*, *Muribaculaceae*, *Quadrisphaera*, CAG_352, *Morganella*, *Bacteroides*, *Sellimonas*, *Romboutsia*, *Collinsella,* and *Jeotgalicoccus* were significantly increased in Proximal T, whereas only the abundance of *Helicobacter* was significantly decreased in Proximal T (Fig. 3A, B and Additional file 4: Figure S3B).

### Different microorganisms in proximal T and distal T compared to their respective non tumor samples

Microbiota composition was analyzed by Wilcoxon signed-rank test. From phylum to genus we found many microbes features show contrasting results between proximal and distal gastric cancer samples when compared to their respective non-cancer samples (Additional file 1: Table S2). At phylum level, *Proteobacteria*, *Patescibacteria*, *Other*, *Bdellovibrionota* and *Fusobacteriota* were significantly increased in Distal T, wheres *Spirochaetota* was significantly decreased in Distal T. However, these microbiota were no significant difference between proximal gastric cancer tumor tissues and control tissues. *Myxococcota* and *Cyanobacteria* were significantly increased in Proximal T, wheres *Acidobacteriota* was significantly decreased in Proximal T. These three microorganisms were no significantly difference between Distal T and Distal N. Compared with the results of LEfSe analysis, the differential microorganisms that distinguish proximal gastric cancer from distal gastric cancer are marked in the table with asterisks.

### Differences in the metabolome profiles between distal T and distal N

**Fig. 4:**
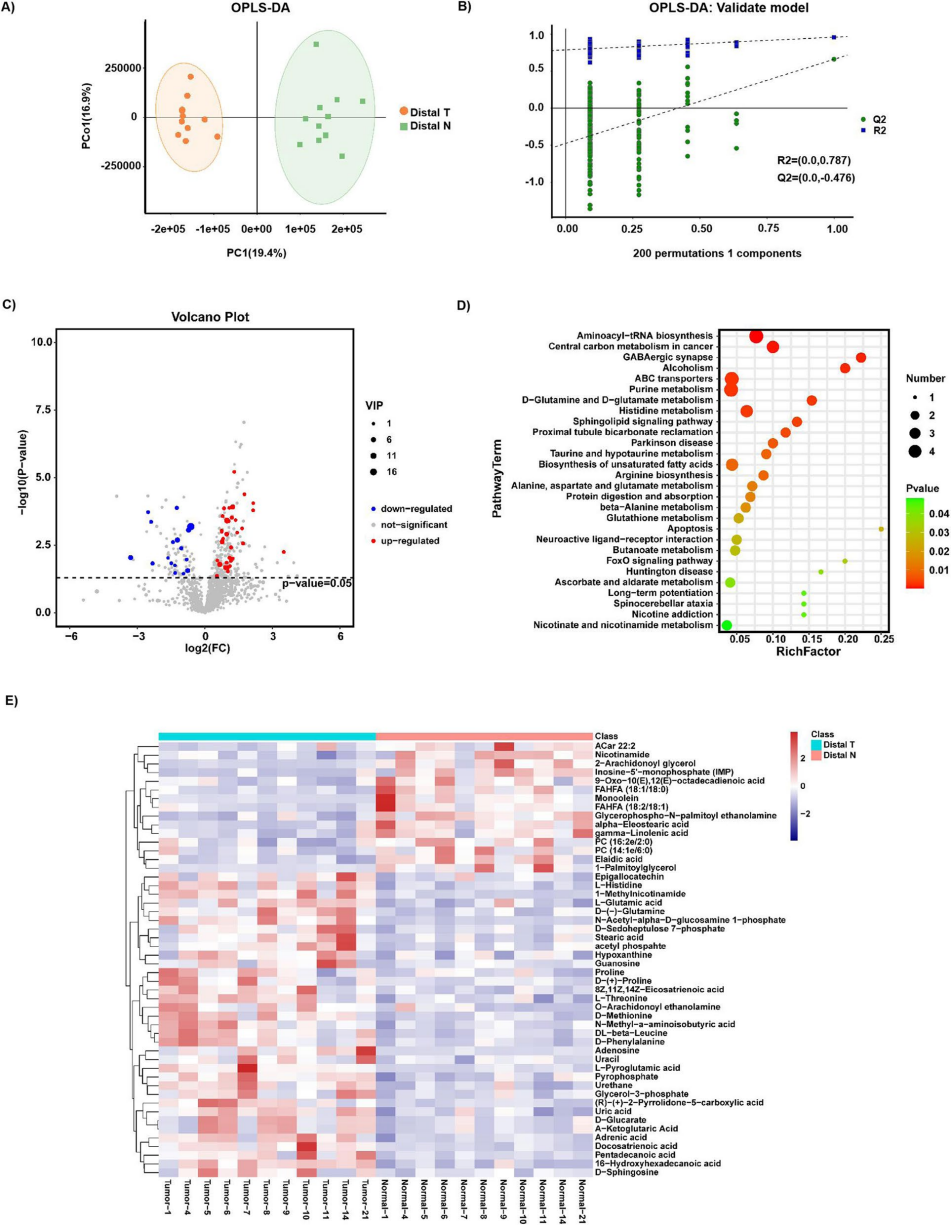
Metabolite composition and difference between Distal T and Distal N. **A**, **B** OPLS-DA showed that Distal T and Distal N were separated into two clusters. Test for OPLS-DA model showed that the model for this study was valid. **C** Volcano map of different metabolites between Distal T and Distal N. (VIP > 1 and p value < 0.05). **D** The functions of these metabolites and metabolic pathways were studied using the KEGG database. **E** Heatmap representative differentially metabolites between Distal T and Distal N. Tumor, represents the samples of Distal T; Normal, represents the samples of Distal N.The abscissa represents the sample name and the ordinate represents the differential metabolite. The color from blue to red indicates that the expression abundance of metabolites is from low to high, that is, the more red indicates that the expression abundance of differential metabolites is higher

Given the diversity and composition of the gastric microbiota were different in Distal T and Distal N, we hypothesized that changes in the metabolome pathways could be partially influenced by the gastric microbiota of the patients. Thus, untargeted metabolomics analysis of the samples (11 Distal T and 11 Distal N) was performed using UHPLC-MS/MS, and 1207 metabolites were quantified in the positive and negative modes. The OPLS-DA score plot illustrated that Distal T and Distal N were separated into two distinct clusters (R2Y = 0.958 and R2X = 0.453) (Fig. 4A). The test for the OPLS-DA model illustrated that the R2 value was larger than the Q2 value and that the Q2 regression line had a negative intercept (R2 = [0.0, 0.787], Q2 = [0.0, -0.476]), indicating that the OPLS-DA model for this study was valid (Fig. 4B). Volcanic maps were used to depict 54 metabolites with significant differential relative abundance between the Distal T and Distal N (VIP > 1 and p-value < 0.05) (Fig. 4C). Compared to Distal N, 17 differential metabolites were down-regulated in Distal T, and 37 were up-regulated. After p-value calibration by the Bonferroni method, 30 significantly different metabolites were identified between Distal T and Distal N (Additional file 1: Table S3). Hierarchical clustering of the top 50 significantly differential metabolites (Fig. 4E) was performed to explore the expression and relationship of the different metabolites between different samples. In order to determine the main metabolic pathways and signal pathways related to the differential metabolites in Distal T and Distal N, KEGG enrichment analysis was performed. Figure 4D illustrates the discriminative metabolites scattered through multiple pathways, including aminoacyl-tRNA biosynthesis, central carbon metabolism in cancer, GABAergic synapse, alcoholism, ABC transporters, histidine metabolism, biosynthesis of unsaturated fatty acids, protein digestion and absorption, glutathione metabolism, etc. Compared to the metabolites of Proximal T related pathways, the enrichment pathways of Distal T included purine metabolism, D-glutamine, and D-glutamate metabolism, sphingolipid signaling pathway, taurine and hypotaurine metabolism, arginine biosynthesis, alanine, aspartate and glutamate metabolism, β-alanine metabolism, butanoate metabolism, ascorbate and aldarate metabolism, and nicotinate and nicotinamide metabolism (Fig. 4D and Additional file 1: Table S5).

### Differences in the metabolome profiles between proximal T and proximal N

**Fig. 5:**
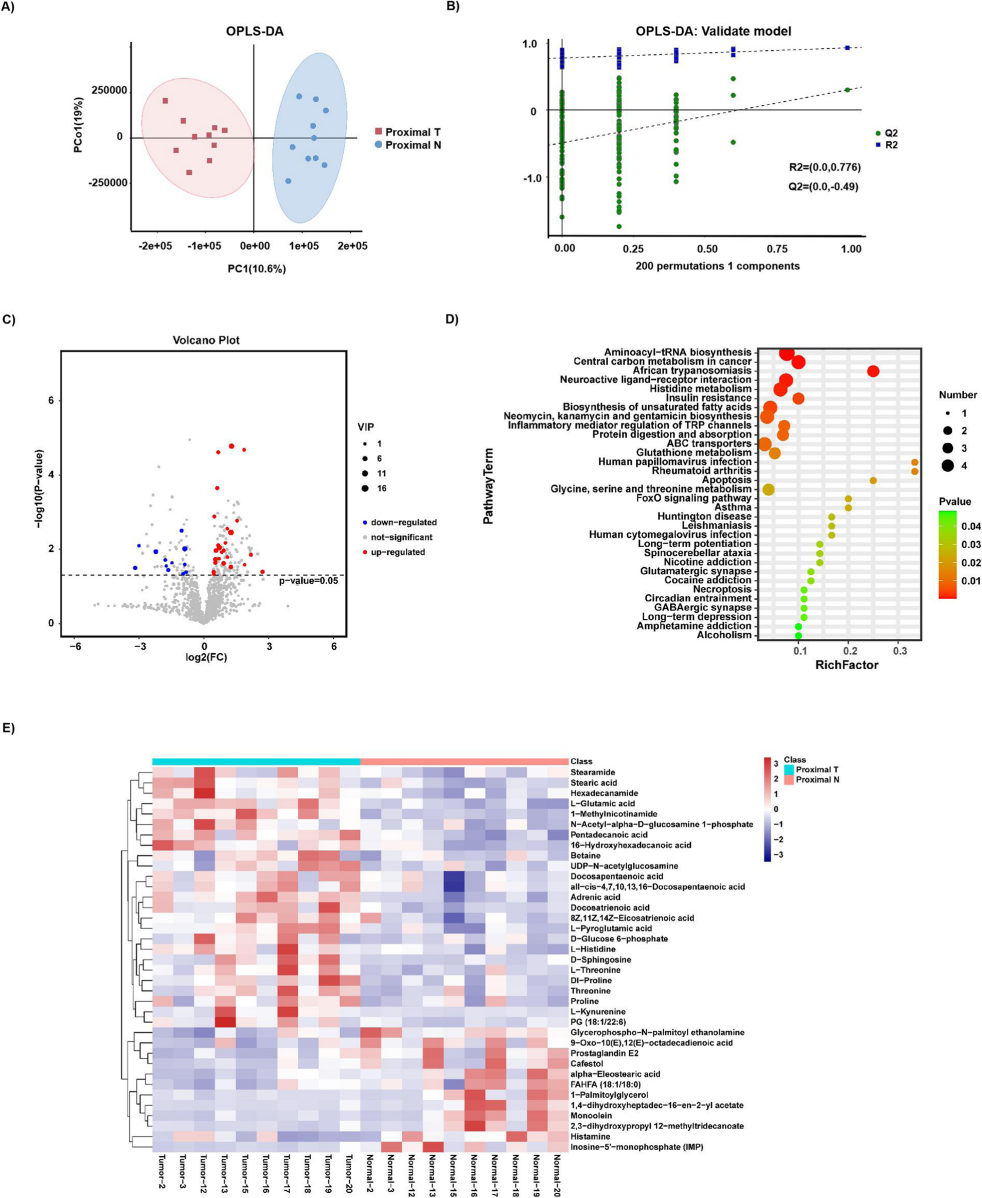
Metabolite composition and difference between Proximal T and Proximal N. **A**, **B** OPLS-DA showed that Proximal T and Proximal N were separated into two clusters. Test for OPLS-DA model showed that the OPLS-DA model for this study was valid. **C** Volcano map of different metabolites between Proximal T and Proximal N. (VIP > 1 and p value < 0.05). **D** The functions of these metabolites and metabolic pathways were studied using the KEGG database. **E** Heatmap representative differentially metabolites between Proximal T and Proximal N. Tumor, represents the samples of Proximal T; Normal, represents the samples of Proximal N.The abscissa represents the sample name and the ordinate represents the differential metabolite. The color from blue to red indicates that the expression abundance of metabolites is from low to high, that is, the more red indicates that the expression abundance of differential metabolites is higher

Likewise, untargeted metabolomics analysis of the samples (10 Proximal T and Proximal N) was also performed using UHPLC-MS/MS. The OPLS-DA score plot delineated that Proximal T and Proximal N were separated into two distinct clusters (R2Y = 0.928 and R2X = 0.438) (Fig. 5A). The test for the OPLS-DA model showed that the R2 value was larger than the Q2 value and that the Q2 regression line had a negative intercept (R2 = [0.0, 0.776], Q2 = [0.0, -0.49]), signaling that the OPLS-DA model for this study was valid (Fig. 5B). Volcanic maps depicted 37 significantly different metabolites between Proximal T and Proximal N (VIP > 1 and p-value < 0.05) (Fig. 5C). 12 differential metabolites were down-regulated in Proximal T, and 25 differential metabolites were up-regulated in Proximal T. Following p-value calibration by the Bonferroni method, 4 significantly different metabolites were discovered between Proximal T and Proximal N (Additional file 1: Table S4). KEGG analysis determined that discriminative metabolites scattered through multiple pathways, like the pathways in distal GC, also included aminoacyl-tRNA biosynthesis, central carbon metabolism in cancer, neuroactive ligand-receptor interaction, histidine metabolism, protein digestion and absorption, ABC transporters, glutathione metabolism, etc. Compared to the metabolites of Distal T related pathways, the enrichment pathways of Proximal T included insulin resistance, inflammatory mediator regulation of TRP channels, glycine, serine and threonine metabolism, circadian entrainment, etc. (Fig. 5E and Additional file 1: Table S6). A heatmap representative of differential metabolites between Proximal T and Proximal N was generated (Fig. 5E).

### Different metabolites in proximal T and distal T compared to their respective non-tumor samples

Compared with proximal gastric cancer, there were more differential metabolites between Distal T and Distal N. All the differential metabolites of Proximal T and Proximal N were included in the differential metabolites of Distal T and Distal N. In contrast, the abundance of adrenic acid, 1-methylnicotinamide, L-glutamic acid, 8Z,11Z,14Z-eicosatrienoic acid, and pentadecanoic acid were significantly elevated in Distal T (Additional file 1: Table S3 and Table S4).The OPLS-DA score plot delineated that Distal N and Proximal N were not divided into two clusters(R2Y = 0.8 and R2X = 0.477) (Additional file 5: Figure S4 A and B).

### The relationship between discriminative genera and metabolites in different genera

**Fig. 6:**
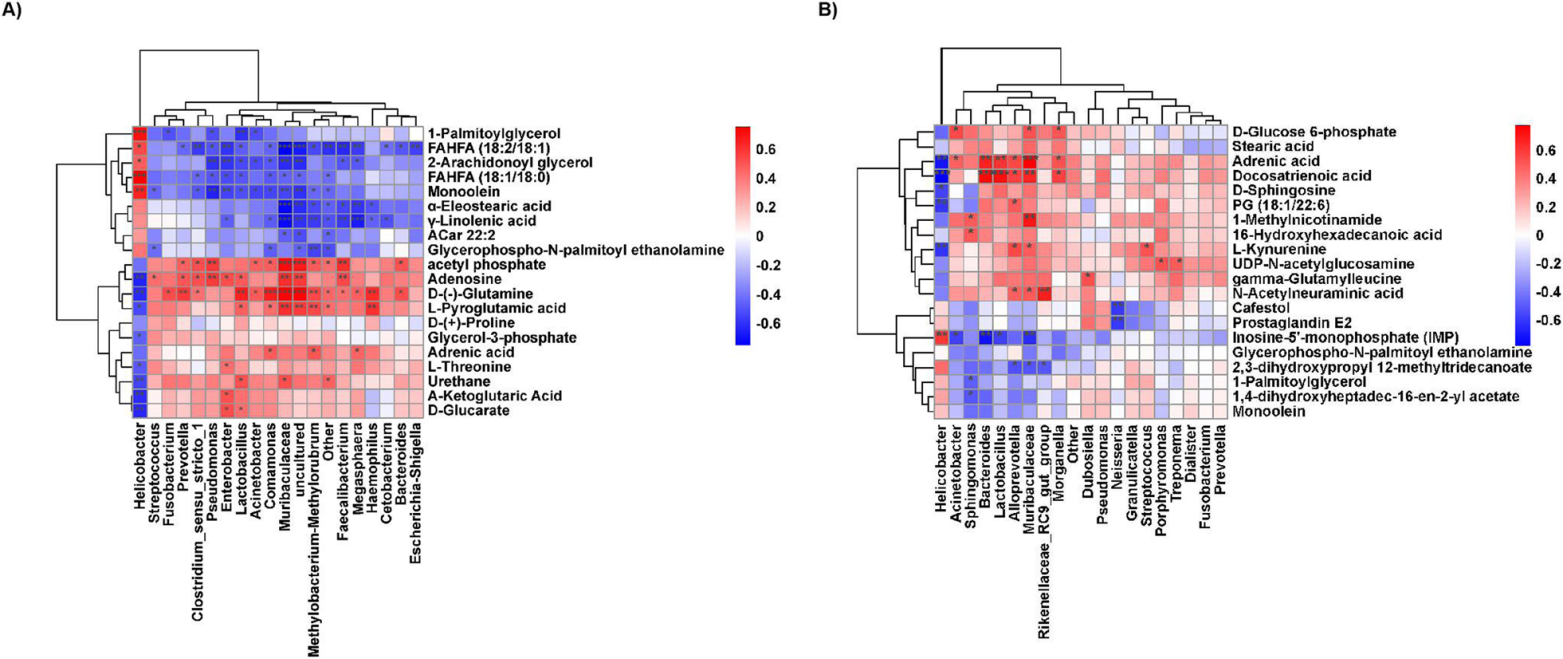
The integrated analysis of microbiota and metabolites. The association between top20 genera and 20 differential metabolites were analyzed using the Spearman’s correlation method. **A** Distal T vs Distal N, **B** Proximal T vs Proximal N. The abscissa represents differential microorganisms and the ordinate represents differential metabolites. Red, positive correlations; Blue, negative correlations. Darker the color, indicating that the correlation is more significant.*p < 0.05; **p < 0.01;***p < 0.001

Spearman’s correlation analysis was employed to assess the association between differential genera and discriminative metabolites in the main enriched pathways (Fig. 6). In distal and proximal GCs, non-tumor tissues were enriched with *Helicobacter*. *Helicobacter* had a significantly positive correlation with fatty acid metabolism but a significantly negative correlation with amino acid metabolism and glucose metabolism. The abundance of *Streptococcus*, *Prevotella*, *Enterobacter*, *Lactobacillus*, *Acinetobacter*, *Muribaculaceae*, *Methylobacterium-Methylorubrum*, and *Faecalibacterium* were significantly increased in Distal T and positively correlated with all the differential metabolites in the glutathione, purine, and multiple amino acids metabolism pathways. These bacteria had a significantly negative correlation with fatty acid metabolism (Fig. 6A). The abundance of *Bacteroides*, *Lactobacillus*, *Muribaculaceae*, *Rikenellaceae_RC9_gut_group*, and *Morganella* were significantly increased in Proximal T and positively correlated with amino acid metabolic pathway and glucose metabolism. However, they were negatively correlated with fatty acid metabolism. Interestingly, *Muribaculaceae* also had a significantly positive correlation with N-Acetylneuraminic acid and 1-Methylnicotinamide. In contrast, *Rikenellaceae_RC9_gut_group* was positively correlated with 1-Methylnicotinamide (Fig. 6B).

## Discussion

To the best of our knowledge, this is the first study to explore the differences in microbial diversity between proximal and distal GCs. Herein, the diversity and richness of the gastric microbiota were not significantly different between Proximal T and Distal T. At the phylum level, *Campilobacterota* was significantly decreased in Proximal T and Distal T. *Bacteroidota*, *Firmicutes*, *Actinobacteriota,* and *Desulfobacterota* were increased in Distal T and Proximal T. *Acidobacteriota*, *Myxococcota* and *Cyanobacteria* were increased in Proximal T. *Proteobacteria*, *Fusobacteriota*, *Spirochaetota*, *Patescibacteria,* and *Bdellovibrionota* were significantly increased in Distal T. At the genus level, *Helicobacter* was decreased in Proximal T and Distal T. This result is consistent with the previous results of other researchers [22]. *Lactobacillus* and *Muribaculaceae* were both increased in Distal T and Proximal T. Sonveaux et al. reported that *Lactobacillus* might generate metabolites that could be used as an energy source for tumor growth and angiogenesis [23]. Zhang et al. proposed that since the abundance of *Muribaculaceae* was increased in cholangiocarcinoma, it could be a promising biomarker for its diagnosis [24]. However, *Prevotella*, *Streptococcus*, *Acinetobacter*, *Faecalibacterium*, *Enterobacter*, *Methylobacterium-Methylorubrum,* and *Alloprevotella* were increased only in Distal T. In comparison, *Rikenellaceae_RC-_gut_group*, *Methylophilus*, *Bacteroides*, *Morganella*, *Romboutsia*, *Parabacteroides,* and *Desulfovibrio* were only increased in Proximal T. When Distal T and Proximal T were compared, the abundance of *Methylobacterium-Methylorubrum* was increased in Distal T, whereas that of *Rikenellaceae_RC9_gut_group* was significantly increased in Proximal T at the genus level. Limited studies on *Methylobacterium-Methylorubrum* suggested that it could survive in extreme environments and was related to drug resistance [25, 26]. Previous studies have also evinced that *Rikenellaceae_RC9_gut_group* is associated with inflammation [27].

The metabolome analysis of Distal T and Distal N revealed 54 significant differential metabolites. However, the comparison between Proximal T and Proximal N revealed merely 37 metabolites with significant differences. KEGG analysis demonstrated that these different metabolites of distal gastric cancer and proximal gastric cancer were related to aminoacyl-tRNA biosynthesis, central carbon metabolism in cancer, neuroactive ligand-receptor interaction, histidine metabolism, biosynthesis of unsaturated fatty acids, protein digestion and absorption, ABC transporters, glutathione metabolism, apoptosis, FoxO signaling pathway, Huntington disease, long-term potentiation, spinocerebellar ataxia, nicotine addiction, GABAergic synapse, and alcoholism. The results are in line with the findings of previous studies. Gao et al. also determined that the aminoacyl tRNA biosynthesis pathway in GC tissues was significantly up-regulated compared with adjacent non-tumor tissues [28]. The neuroactive live receptor interaction pathway is involved in the tumor microenvironment and cell-cell communication [29]. The relative abundance of the differential metabolites of amino acids in tumor tissues was higher than in non-tumor tissues. A study by Tsai et al. unveiled the aberrant metabolism of multiple amino acids in gastric cancer [30]. The relative abundance of the differential metabolites of amino acids in tumor tissues was higher than in non-tumor tissues. Since tumor cells use amino acids to produce energy and synthesize proteins and nucleosides, increasing the concentration of amino acids is essential for tumor cell proliferation.

Different metabolites in distal GC were also correlated with purine metabolism, D-glutamine and D-glutamate metabolism, sphingolipid signaling pathway, proximal tubule bicarbonate reclamation, Parkinson’s disease, taurine and hypotaurine metabolism, arginine biosynthesis, alanine, aspartate and glutamate metabolism, β-alanine metabolism, butanoate metabolism, ascorbate, and aldarate metabolism, and nicotinate and nicotinamide metabolism. Prior studies have highlighted that the sphingolipid signaling pathway, alcoholism, glutathione metabolism, taurine and hypotaurine metabolism, alanine, aspartate, and glutamate metabolism were all associated with GC [31–34]. Strikingly, PD has been associated with most cancers in Taiwan [35]. Abnormal arginine metabolism is a potential treatment for GC [36]. Previous studies have also shown that the GC differential genes are enriched in the butyric acid metabolic pathway [37]. Taylor et al. concluded that the ascorbic acid pathway was related to melanoma [38]. Cumulative evidence suggests that nicotinamide plays an instrumental role in cancer prevention and therapy [39]. Interestingly, the abundance of nicotinamide related metabolites was decreased in distal GC. Moreover, different metabolites identified in proximal GC were also related to insulin resistance, inflammatory mediator regulation of TRP channels, human papillomavirus infection, rheumatoid arthritis, glycine, serine, and threonine metabolism, glutamatergic synapse, cocaine addiction, necroptosis, circadian entrainment, and so on. Kwon et al. hypothesized that insulin resistance could be an independent risk factor for GC [40]. Studies have corroborated that human cytomegalovirus infection is related to the occurrence and development of GC [41]. The transient receptor potential (TRP) channel is the key receptor of pain stimulation signal transduction. The substances released by the microenvironment of different types of cancer govern the activity of TRPs by regulating intracellular signaling pathways [42]. Prolonged immune dysregulation and the resulting inflammatory response associated with the development of rheumatoid arthritis could also lead to increased cancer development risk [43]. There are overwhelming reports supporting the role of supplementary glycine in the prevention of many diseases and disorders, including cancer [44]. Circadian timing can modify 2- to tenfold the tolerability of anticancer medications in experimental models and cancer patients [45]. A study of colorectal cancer exposed the differences in genes between the liver metastases and the primary tumors. KEGG analysis of mutant genes showed that the mutations were mainly distributed in circadian entrainment, insulin secretion, and glutamatergic synapses [46]. These results indicate that the occurrence of distal GC may be closely related to the disorder of amino acid metabolism, lipid metabolism, and nucleotide metabolism. Additionally, the occurrence and development of proximal GC may be related to hormone dysregulation.

According to Spearman’s correlation analysis, *Methylobacterium-Methylorubrum,* which was significantly increased in Distal T, was positively correlated with adrenic acid, L-pyroglutamic acid, D-(-)-glutamine, and acetyl phosphate. However, *Methylobacterium-Methylorubrum* was negatively correlated with glycerophospho-N-palmitoyl ethanolamine, γ-linolenic acid, α-eleostearic acid, monoolein, and FAHFA. L-pyroglutamic acid and glutamine are metabolites of glutamine metabolism, and they were significantly increased in Distal T[47]. Studies have found that *Streptococcus* can metabolize glutamine into pyroglutamate and ammonia [48]. Recent research implies that adrenic acid can determine the sensitivity of ferroptosis in gastric cancer [49]. Duan et al. showed that glycosphospho-N-palmitoyl ethanolamine is a potential biomarker of depression [50]. Besides, correlation analysis revealed that *Methylobacterium-Methylorubrum* was associated with the decrease in glycosphospho-N-palmitoyl ethanolamine in Distal T. γ-linolenic acid can inhibit the growth and epithelial-mesenchymal transformation of gastric cancer cells [51]. Meanwhile, α-Eleostearic acid inhibits the proliferation of breast cancer cells [52]. Monoolein and nanocomposites could be utilized for cancer drug delivery [53]. Some FAHFAs can enhance glucose tolerance and insulin sensitivity, stimulate insulin secretion, and exert anti-inflammatory effects [54]. *Rikenellaceae_RC_gut_group,* which was significantly increased in Proximal T, was positively correlated with N-acetylneuraminic acid, also referred to as sialic acid, and negatively correlated with 2,3-dihydroxypropyl 12-methyltridecanoate. Büll et al. observed that sialic acid had a key role in tumor immune escape. They proved that sialic acid block creates an immune permissive tumor microenvironment for CD8 + T cell-mediated tumor immunity [55]. Earlier studies have reported that *Legionella pneumophila* can be metabolized to 2,3-dihydroxypropyl 12-methyltridecanoate [56]. The aforementioned results showed differences in microbial-related metabolites in proximal and distal GCs, which further suggested that there are distinct mechanisms for the occurrence and development of proximal and distal GCs.

Our study had several limitations that need to be taken into account. First, the sample size of proximal and distal GCs was limited, resulting in no significant difference in the microbial diversity and abundance between proximal and distal GCs. Second, differential microbiota and metabolites were discovered in proximal and distal GCs, and the pathways associated with metabolite enrichment were preliminarily analyzed. Nevertheless, no further experiments have been undertaken to determine the cause of this difference. Lastly, dietary habits and medications impact microbial composition and metabolism, but the patients’ dietary and medication data were not collected.

In conclusion, to the best of our knowledge, the differences in microbial composition and metabolites in proximal and distal GCs were described for the first time, and the correlation between microbiota and metabolites was preliminarily discussed. *Methylobacterium-Methylorubrum* was significantly increased in Distal T, positively correlated with cancer-promoting metabolites, and negatively correlated with cancer-inhibiting metabolites. Moreover, *Rikenellaceae_RC_gut_group* was significantly increased in Proximal T and was positively correlated with cancer-promoting metabolites. These metabolites and microbiota could be related to the various mechanisms involved in the occurrence and development of proximal and distal GCs and provide a foundation for future treatments; hence, they deserve further study.

## Supplementary Information

**Additional file 1: Table S1.** Clinicopathological characteristics of patients with gastric cancer in this study. **Table S2.** Different microorganisms in Proximal T and Distal T compared to their respective non tumor samples. **Table S3.** Significant differences in metabolites between Distal T and Distal N. **Table S4.** Significant differences in metabolites between Proximal T and Proximal N. **Table S5.** Metabolic pathway enrichment of differential metabolites between Distal T and Distal N. **Table S6.** Metabolic pathway enrichment of differential metabolites between Proximal T and Proximal N.

**Additional file 2: Figure S2.** The microbial relative abundance of proximal, distal tumor tissues and matched non-tumor tissues at the genus level. Proximal T,proximal GC tumor tissues; Proximal N,proximal GC non-tumor tissues; Distal T, distal GC tumor tissues; Distal N, distal GC non-tumor tissues.

**Additional file 3: Figure S2.** The microbial relative abundance of proximal, distal tumor tissues and matched non-tumor tissues at the genus level. Proximal T,proximal GC tumor tissues; Proximal N,proximal GC non-tumor tissues; Distal T, distal GC tumor tissues; Distal N, distal GC non-tumor tissues.

**Additional file 4: Figure S3.** Differential microbiota of gastric cancer tissues and non-tumor tissues. **A** Differential taxa from phylum to genus of Distal T and Distal N. **B** Differential taxa from phylum to genus of Proximal T and Proximal N.

**Additional file 5: Figure S4.** Metabolite composition and difference between Proximal N and Distal N. **A**, **B** OPLS-DA showed that Proximal N and Distal N were not separated into two clusters. Test for OPLS-DA model showed that the OPLS-DA model for this study was valid.

## Data Availability

Thank you for your advise. Raw sequence data of 16S RNA microbiome have been uploaded to China National Microbiological Data Center (Project accession number NMDC10017675). The corresponding author has access to all data in the study.
