# Supplementary material for: Microbiota and metabolites alterations in proximal and distal gastric cancer patients
Source: J Transl Med. 2022 Sep 30;20:439. doi: 10.1186/s12967-022-03650-x (PMC9524040; doi:10.1186/s12967-022-03650-x)
Supplement: Supplementary file 1 — Additional file 1: Table S1. Clinicopathological characteristics of patients with gastric cancer in this study. Table S2. Different microorganisms in Proximal T and Distal T compared to their respective non tumor samples. Table S3. Significant differences in metabolites between Distal T and Distal N. Table S4. Significant differences in metabolites between Proximal T and Proximal N. Table S5. Metabolic pathway enrichment of differential metabolites between Distal T and Distal N. Table S6. Metabolic pathway enrichment of differential metabolites between Proximal T and Proximal N. [file 12967_2022_3650_MOESM1_ESM.docx]

**Supplementary Data**

**Table S1 Clinicopathological characteristics of patients with gastric cancer in this study**

| **Characteristics** | **Proximal** | **Distal** |
| --- | --- | --- |
| Total number | 13 | 16 |
| Gender, no |  |  |
| Female | 3 | 4 |
| Male | 10 | 12 |
| Age (means ± SD) | 68.38461538±7.47 | 61.56±6.31 |
| Weight (Kg, means ± SD) | 59.5±8.74 | 62.27±8.43 |
| Height (cm, means ± SD) | 165.4±6.75 | 164.2±7.20 |
| BMI (means ± SD) | 22.24±2.64 | 23.06±2.51 |
| Complications, no |  |  |
| Hypertension | 3 | 2 |
| Diabetes mellitus | 0 | 2 |
| Tumor differentiation, no |  |  |
| Moderately differentiated | 2 | 4 |
| Moderately-poorly differentiated | 4 | 5 |
| Poorly differentiated | 7 | 7 |
| Lauren typing, no |  |  |
| Intestinal type | 7 | 12 |
| Diffuse type | 1 | 1 |
| Mixed type | 5 | 3 |
| Tumor stage, no |  |  |
| I | 2 | 3 |
| II | 7 | 8 |
| III | 4 | 5 |

**Table S2 Different microorganisms in Proximal T and Distal T compared to their respective non tumor samples**

| **Microorganisms** | **P value** | |
| --- | --- | --- |
|  | **Distal T vs Distal N** | **Proximal T vs Proximal N** |
| **Phylum** | | |
| **Proteobacteria* | 0.008991009 | NS |
| **Patescibacteria* | 0.00999001 | NS |
| **Other* | 0.026973027 | NS |
| *Bdellovibrionota* | 0.030969031 | NS |
| *Spirochaetota* | 0.040959041 | NS |
| *Fusobacteriota* | 0.048951049 | NS |
| *Acidobacteriota* | NS | 0.003996004 |
| *Myxococcota* | NS | 0.031968032 |
| *Cyanobacteria* | NS | 0.032967033 |
| **Class** | | |
| **Alphaproteobacteria* | 0.004995005 | NS |
| **Gammaproteobacteria* | 0.005994006 | NS |
| **Negativicutes* | 0.018981019 | NS |
| *Ktedonobacteria* | 0.021978022 | NS |
| **Bacilli* | 0.023976024 | NS |
| *Spirochaetia* | 0.033966034 | NS |
| **Other* | 0.040959041 | NS |
| *Subgroup_22* | 0.048951049 | NS |
| *Fusobacteriia* | 0.04995005 | NS |
| *Acidobacteriae* | NS | 0.006993007 |
| *AD3* | NS | 0.02997003 |
| **Actinobacteria* | NS | 0.031968032 |
| *Cyanobacteriia* | NS | 0.037962038 |
| *Thermoleophilia* | NS | 0.03996004 |
| *Deferribacteres* | NS | 0.044955045 |
| **Order** | | |
| **Burkholderiales* | 0.002997003 | NS |
| *Mycoplasmatales* | 0.005994006 | NS |
| **Rhizobiales* | 0.012987013 | NS |
| **Caulobacterales* | 0.013986014 | NS |
| **Bacteroidales* | 0.01998002 | NS |
| **Lactobacillales* | 0.01998002 | NS |
| *Elev-16S-1166* | 0.022977023 | NS |
| **Veillonellales-Selenomonadales* | 0.025974026 | NS |
| *Spirochaetales* | 0.026973027 | NS |
| **Other* | 0.02997003 | NS |
| *Thermomicrobiales* | 0.02997003 | NS |
| *Geobacterales* | 0.033966034 | NS |
| *bacteriap25* | 0.036963037 | NS |
| *Sphingomonadales* | 0.038961039 | NS |
| *SBR1031* | 0.040959041 | NS |
| *Subgroup_22* | 0.043956044 | NS |
| *Nannocystales* | 0.045954046 | NS |
| *Bifidobacteriales* | NS | 0.000999001 |
| *Clostridia_UCG-014* | NS | 0.002997003 |
| **Enterobacterales* | NS | 0.003996004 |
| *Acidobacteriales* | NS | 0.003996004 |
| *Micromonosporales* | NS | 0.012987013 |
| *Kineosporiales* | NS | 0.012987013 |
| *Clostridiales* | NS | 0.014985015 |
| *Subgroup_2* | NS | 0.01998002 |
| *AD3* | NS | 0.020979021 |
| *Acidaminococcales* | NS | 0.021978022 |
| *Actinomarinales* | NS | 0.033966034 |
| *Vibrionales* | NS | 0.033966034 |
| *Deferribacterales* | NS | 0.040959041 |
| *Christensenellales* | NS | 0.044955045 |
| *Paracaedibacterales* | NS | 0.044955045 |
| *Rhodospirillales* | NS | 0.045954046 |
| *Acetobacterales* | NS | 0.047952048 |
| *Ferrovibrionales* | NS | 0.04995005 |
| *Solibacterales* | NS | 0.04995005 |
| **Family** | | |
| **Beijerinckiaceae* | 0.000999001 | NS |
| *Selenomonadaceae* | 0.002997003 | NS |
| *Mycoplasmataceae* | 0.003996004 | NS |
| *Paludibacteraceae* | 0.003996004 | NS |
| *Hyphomicrobiaceae* | 0.00999001 | NS |
| *Aerococcaceae* | 0.010989011 | NS |
| **Caulobacteraceae* | 0.011988012 | NS |
| *Methylococcaceae* | 0.011988012 | NS |
| *Alcanivoracaceae1* | 0.012987013 | NS |
| *Peptostreptococcaceae* | 0.013986014 | NS |
| *Lentimicrobiaceae* | 0.014985015 | NS |
| *Amb-16S-1323* | 0.015984016 | NS |
| *Elev-16S-1166* | 0.018981019 | NS |
| *Fibrobacteraceae* | 0.018981019 | NS |
| **SM2D12* | 0.022977023 | NS |
| **Rhizobiales_Incertae_Sedis* | 0.023976024 | NS |
| *SBR1031* | 0.025974026 | NS |
| *Rhizobiaceae* | 0.026973027 | NS |
| **Streptococcaceae* | 0.028971029 | NS |
| *Geobacteraceae* | 0.028971029 | NS |
| *JG30-KF-CM45* | 0.028971029 | NS |
| *Fusobacteriaceae* | 0.034965035 | NS |
| **Prevotellaceae* | 0.037962038 | NS |
| *Yersiniaceae* | 0.042957043 | NS |
| *AKYH767* | 0.042957043 | NS |
| *Spirochaetaceae* | 0.045954046 | NS |
| *Moraxellaceae* | 0.047952048 | NS |
| *Thioglobaceae* | 0.048951049 | NS |
| *bacteriap25* | 0.04995005 | NS |
| *Bifidobacteriaceae* | NS | 0.000999001 |
| *Erysipelatoclostridiaceae* | NS | 0.000999001 |
| *uncultured* | NS | 0.001998002 |
| *Enterobacteriaceae* | NS | 0.002997003 |
| **Morganellaceae* | NS | 0.003996004 |
| *Clostridia_UCG-014* | NS | 0.003996004 |
| *Sutterellaceae* | NS | 0.004995005 |
| *Marinifilaceae* | NS | 0.006993007 |
| *[Eubacterium]_coprostanoligenes_group* | NS | 0.007992008 |
| **Bacteroidaceae* | NS | 0.00999001 |
| *Clostridiaceae* | NS | 0.010989011 |
| *Enterococcaceae* | NS | 0.012987013 |
| *Micromonosporaceae* | NS | 0.013986014 |
| *Kineosporiaceae* | NS | 0.013986014 |
| *Bacillaceae* | NS | 0.014985015 |
| *Eggerthellaceae* | NS | 0.014985015 |
| *Acidaminococcaceae* | NS | 0.017982018 |
| *UCG-010* | NS | 0.01998002 |
| *AD3* | NS | 0.020979021 |
| *Leuconostocaceae* | NS | 0.022977023 |
| *Rhodocyclaceae* | NS | 0.023976024 |
| *Pseudoalteromonadaceae* | NS | 0.027972028 |
| *Subgroup_2* | NS | 0.031968032 |
| *Vagococcaceae* | NS | 0.036963037 |
| *Kiloniellaceae* | NS | 0.038961039 |
| *Akkermansiaceae* | NS | 0.040959041 |
| *Vibrionaceae* | NS | 0.040959041 |
| *Desulfomicrobiaceae* | NS | 0.041958042 |
| *Deferribacteraceae* | NS | 0.043956044 |
| **Coriobacteriaceae* | NS | 0.043956044 |
| *Rs-E47_termite_group* | NS | 0.043956044 |
| *Acidobacteriaceae_(Subgroup_1)* | NS | 0.045954046 |
| *Christensenellaceae* | NS | 0.047952048 |
| *Sulfurimonadaceae* | NS | 0.048951049 |
| **Genus** | | |
| *Methylocaldum* | 0.001998002 | NS |
| *Mycoplasma* | 0.002997003 | NS |
| *Shuttleworthia* | 0.002997003 | NS |
| *Paludibacter* | 0.003996004 | NS |
| *Allisonella* | 0.003996004 | NS |
| *Aerococcus* | 0.005994006 | NS |
| **Methylobacterium-Methylorubrum* | 0.007992008 | NS |
| **Alloprevotella* | 0.007992008 | NS |
| *Alcanivorax* | 0.00999001 | NS |
| *Hyphomicrobium* | 0.00999001 | NS |
| *Colidextribacter* | 0.010989011 | NS |
| *[Eubacterium]_hallii_group* | 0.010989011 | NS |
| *Amb-16S-1323* | 0.012987013 | NS |
| *Kurthia* | 0.014985015 | NS |
| **Atopobium* | 0.015984016 | NS |
| *Anaeroplasma* | 0.015984016 | NS |
| **Streptococcus* | 0.018981019 | NS |
| *Lentimicrobium* | 0.018981019 | NS |
| *Prevotellaceae_UCG-001* | 0.018981019 | NS |
| *JG30-KF-CM45* | 0.020979021 | NS |
| *Centipeda* | 0.021978022 | NS |
| *Citrifermentans* | 0.022977023 | NS |
| *Terrimonas* | 0.022977023 | NS |
| *Intestinibacter* | 0.025974026 | NS |
| *CL500-29_marine_group* | 0.026973027 | NS |
| *Sphingomonas* | 0.027972028 | NS |
| *Fusicatenibacter* | 0.028971029 | NS |
| *Edaphobaculum* | 0.028971029 | NS |
| *Abiotrophia* | 0.02997003 | NS |
| *Elev-16S-1166* | 0.030969031 | NS |
| **Psychrobacter* | 0.031968032 | NS |
| *Solobacterium* | 0.033966034 | NS |
| *SBR1031* | 0.033966034 | NS |
| **SM2D12* | 0.033966034 | NS |
| *Anaerostipes* | 0.035964036 | NS |
| *Solirubrobacter* | 0.037962038 | NS |
| *Selenomonas* | 0.037962038 | NS |
| *Alteromonas* | 0.037962038 | NS |
| *Archangium* | 0.038961039 | NS |
| *Fibrobacter* | 0.038961039 | NS |
| *bacteriap25* | 0.040959041 | NS |
| *Oscillospira* | 0.040959041 | NS |
| *Acinetobacter* | 0.041958042 | NS |
| *Soonwooa* | 0.041958042 | NS |
| *Prevotella* | 0.043956044 | NS |
| *Marmoricola* | 0.043956044 | NS |
| *Serratia* | 0.044955045 | NS |
| *Subgroup_22* | 0.047952048 | NS |
| *[Eubacterium]_nodatum_group* | 0.04995005 | NS |
| *Sporosarcina* | 0.04995005 | NS |
| *Subdoligranulum* | NS | 0.000999001 |
| *Klebsiella* | NS | 0.000999001 |
| *Erysipelotrichaceae_UCG-003* | NS | 0.000999001 |
| *Bifidobacterium* | NS | 0.001998002 |
| *Oscillibacter* | NS | 0.001998002 |
| *Enterobacteriaceae* | NS | 0.001998002 |
| *Parasutterella* | NS | 0.002997003 |
| *Rikenella* | NS | 0.002997003 |
| *Lachnospiraceae_UCG-010* | NS | 0.002997003 |
| *ASF356* | NS | 0.002997003 |
| *Pedobacter* | NS | 0.003996004 |
| *Coriobacteriaceae_UCG-002* | NS | 0.004995005 |
| *Holdemanella* | NS | 0.004995005 |
| *Clostridia_UCG-014* | NS | 0.005994006 |
| *Acidovorax* | NS | 0.005994006 |
| **Rikenellaceae_RC9_gut_group* | NS | 0.006993007 |
| *Phascolarctobacterium* | NS | 0.006993007 |
| **Morganella* | NS | 0.007992008 |
| *[Eubacterium]_coprostanoligenes_group* | NS | 0.007992008 |
| *Alcaligenes* | NS | 0.007992008 |
| **Bacteroides* | NS | 0.008991009 |
| *UCG-002* | NS | 0.008991009 |
| *Clostridium_sensu_stricto_1* | NS | 0.00999001 |
| *Erysipelatoclostridium* | NS | 0.00999001 |
| *UBA1819* | NS | 0.00999001 |
| *Enterococcus* | NS | 0.010989011 |
| *Faecalibaculum* | NS | 0.011988012 |
| *Odoribacter* | NS | 0.012987013 |
| *[Eubacterium]_fissicatena_group* | NS | 0.013986014 |
| *Nitratireductor* | NS | 0.013986014 |
| *UCG-010* | NS | 0.014985015 |
| *Anaerotruncus* | NS | 0.015984016 |
| *Enterorhabdus* | NS | 0.017982018 |
| *Subgroup_2* | NS | 0.018981019 |
| **CAG-352* | NS | 0.01998002 |
| *Escherichia-Shigella* | NS | 0.020979021 |
| *Fournierella* | NS | 0.022977023 |
| **Quadrisphaera* | NS | 0.022977023 |
| **Sellimonas* | NS | 0.023976024 |
| *GCA-900066575* | NS | 0.024975025 |
| *Pseudoalteromonas* | NS | 0.025974026 |
| *AD3* | NS | 0.026973027 |
| **Jeotgalicoccus* | NS | 0.026973027 |
| *[Eubacterium]_siraeum_group* | NS | 0.027972028 |
| *Ileibacterium* | NS | 0.028971029 |
| **Romboutsia* | NS | 0.031968032 |
| *Candidatus_Saccharimonas* | NS | 0.032967033 |
| *Vibrio* | NS | 0.034965035 |
| *Agathobacter* | NS | 0.038961039 |
| *Vagococcus* | NS | 0.038961039 |
| *Weissella* | NS | 0.038961039 |
| *Hydrogenophaga* | NS | 0.038961039 |
| *Muribaculum* | NS | 0.03996004 |
| *Akkermansia* | NS | 0.041958042 |
| *Flavonifractor* | NS | 0.041958042 |
| *Dysgonomonas* | NS | 0.041958042 |
| *Buchnera* | NS | 0.041958042 |
| *Tyzzerella* | NS | 0.043956044 |
| *Candidatus_Solibacter* | NS | 0.043956044 |
| **Collinsella* | NS | 0.043956044 |
| *Mitsuokella* | NS | 0.044955045 |
| *Pelagibius* | NS | 0.044955045 |
| *Bacillus* | NS | 0.046953047 |
| *Desulfomicrobium* | NS | 0.047952048 |
| *Allobaculum* | NS | 0.048951049 |
| *UBA6140* | NS | 0.048951049 |
| *Sulfuricurvum* | NS | 0.048951049 |
| *Leuconostoc* | NS | 0.04995005 |

**Blue filling**:indicates that the microorganism was significantly down-regulated in tumor；**Stars**:indicate the differential microorganisms that coincide with the LEfSe analysis.

**Table S3 Significant differences in metabolites between Distal T and Distal N**

| **Metabolites** | **VIP** | **p-value** | **adj.p-value** | **log2(FC)** | **average(Distal T)** | **average(Distal N)** |
| --- | --- | --- | --- | --- | --- | --- |
| *16-Hydroxyhexadecanoic acid | 1.48 | 6.05299E-06 | 0.001089373 | 1.31 | 194467504 | 78580375.2 |
| *N-Acetyl-α-D-glucosamine 1-phosphate | 1.52 | 4.10272E-05 | 0.004126651 | 1.76 | 189390247.3 | 55925391.21 |
| *γ-Glutamylglutamic acid | 1.08 | 8.76021E-05 | 0.005286786 | 2.15 | 98694073.95 | 22240235.31 |
| Adrenic acid | 5.30 | 0.000120065 | 0.006300634 | 1.24 | 2910135177 | 1235518801 |
| *Glycerophospho-N-palmitoyl ethanolamine | 2.75 | 0.00012999 | 0.006300634 | -1.25 | 296479881.4 | 704126487.7 |
| *L-Histidine | 1.90 | 0.000135722 | 0.006300634 | 0.85 | 468235377 | 259692680 |
| *D-Methionine | 1.28 | 0.00013031 | 0.006300634 | 1.09 | 179747060.7 | 84562240.06 |
| 1-Methylnicotinamide | 1.43 | 0.000159792 | 0.007143281 | 2.14 | 162264575.2 | 36698515.51 |
| *Inosine-5'-monophosphate (IMP) | 1.67 | 0.000186937 | 0.008058309 | -2.52 | 32801734.22 | 188086093.4 |
| *Uric acid | 1.68 | 0.00026265 | 0.009703547 | 0.79 | 390646563.1 | 225476517.1 |
| *Urethane | 2.58 | 0.000298511 | 0.010597154 | 1.20 | 679014722.8 | 295912503.8 |
| *DL-β-Leucine | 8.20 | 0.000383191 | 0.011948958 | 0.98 | 9009534836 | 4559567832 |
| *L-Threonine | 1.70 | 0.000403616 | 0.011948958 | 1.06 | 356053751.2 | 170903041.9 |
| *Threonine | 1.03 | 0.000368046 | 0.011948958 | 1.36 | 107512130.2 | 41758493.37 |
| *2-Arachidonoyl glycerol | 2.08 | 0.000428245 | 0.01230695 | -2.39 | 64434372.08 | 337417316.6 |
| *Elaidic acid | 19.23 | 0.000638253 | 0.017119354 | -0.62 | 42224429052 | 65044632984 |
| *(R)-(+)-2-Pyrrolidone-5-carboxylic acid | 1.30 | 0.000751905 | 0.018846592 | 1.66 | 160286576.6 | 50813975.88 |
| *Nicotinamide | 5.91 | 0.00086467 | 0.020070322 | -0.72 | 3397488201 | 5613332921 |
| L-Glutamic acid | 1.85 | 0.000915118 | 0.020840525 | 0.75 | 519964841.9 | 309482718 |
| *Pyrophosphate | 1.47 | 0.000985774 | 0.021633266 | 0.75 | 356027230.9 | 211625824.5 |
| *Docosatrienoic acid | 1.19 | 0.001073263 | 0.022726822 | 1.44 | 142402240.1 | 52630801.21 |
| *D-Phenylalanine | 4.14 | 0.001218454 | 0.0237337 | 0.97 | 2461842992 | 1254579227 |
| *N-Methyl-a-aminoisobutyric acid | 1.30 | 0.001811067 | 0.030788134 | 0.81 | 264126677 | 150563882.8 |
| *α-Eleostearic acid | 7.05 | 0.002021001 | 0.033344767 | -1.22 | 2411373226 | 5612380164 |
| 8Z,11Z,14Z-Eicosatrienoic acid | 5.23 | 0.002345046 | 0.036746843 | 0.77 | 4458108542 | 2614236210 |
| *FAHFA (18:1/18:0) | 2.14 | 0.002405137 | 0.036746843 | -1.42 | 195347998.1 | 521594895.1 |
| Pentadecanoic acid | 1.91 | 0.002460657 | 0.036913488 | 0.75 | 612336770.3 | 363522297.7 |
| *D-(-)-Glutamine | 1.88 | 0.002692507 | 0.039239628 | 1.69 | 354312142.2 | 109499740.3 |
| *L-Pyroglutamic acid | 2.05 | 0.003797848 | 0.046027401 | 1.17 | 545825779.9 | 243381644.6 |
| *γ-Linolenic acid | 2.56 | 0.004049265 | 0.046827507 | -1.05 | 415159445.9 | 859944463.2 |

**Stars**:indicate the different metabolites from proximal gastric cancer.

**Table S4 Significant differences in metabolites between Proximal T and Proximal N**

| **Metabolites** | **VIP** | **p-value** | **adj.p-value** | **log2(FC)** | **average(Proximal T)** | **average(Proximal N)** |
| --- | --- | --- | --- | --- | --- | --- |
| L-Glutamic acid | 2.21 | 2.39693E-05 | 0.007232741 | 0.67 | 470094878.7 | 294927580.9 |
| Adrenic acid | 7.38 | 1.64858E-05 | 0.007232741 | 1.28 | 3054948953 | 1256546035 |
| 1-Methylnicotinamide | 1.67 | 2.05938E-05 | 0.007232741 | 1.87 | 139459602.5 | 38247550.48 |
| Pentadecanoic acid | 2.38 | 0.000222453 | 0.044750203 | 0.62 | 620859001.2 | 402704519.1 |

**Table S5 Metabolic pathway enrichment of differential metabolites between Distal T and Distal N**

| **Annotation** | **in set** | **set** | **in background** | **background** | **RichFactor** | **p-value** |
| --- | --- | --- | --- | --- | --- | --- |
| Aminoacyl-tRNA biosynthesis | 4 | 16 | 52 | 3250 | 0.077 | 9.20758E-05 |
| Central carbon metabolism in cancer | 3 | 16 | 30 | 3250 | 0.100 | 0.000366719 |
| African trypanosomiasis | 2 | 16 | 8 | 3250 | 0.250 | 0.000625518 |
| Neuroactive ligand-receptor interaction | 3 | 16 | 40 | 3250 | 0.075 | 0.000865937 |
| Histidine metabolism | 3 | 16 | 47 | 3250 | 0.064 | 0.001391519 |
| Insulin resistance | 2 | 16 | 20 | 3250 | 0.100 | 0.004100731 |
| Biosynthesis of unsaturated fatty acids | 3 | 16 | 69 | 3250 | 0.043 | 0.004207856 |
| Neomycin, kanamycin and gentamicin biosynthesis | 3 | 16 | 81 | 3250 | 0.037 | 0.006608717 |
| Inflammatory mediator regulation of TRP channels | 2 | 16 | 28 | 3250 | 0.071 | 0.007973157 |
| Protein digestion and absorption | 2 | 16 | 29 | 3250 | 0.069 | 0.008539239 |
| ABC transporters | 3 | 16 | 93 | 3250 | 0.032 | 0.009694149 |
| Glutathione metabolism | 2 | 16 | 38 | 3250 | 0.053 | 0.014409484 |
| Human papillomavirus infection | 1 | 16 | 3 | 3250 | 0.333 | 0.014701142 |
| Rheumatoid arthritis | 1 | 16 | 3 | 3250 | 0.333 | 0.014701142 |
| Apoptosis | 1 | 16 | 4 | 3250 | 0.250 | 0.019556326 |
| Glycine, serine and threonine metabolism | 2 | 16 | 50 | 3250 | 0.040 | 0.024261579 |
| FoxO signaling pathway | 1 | 16 | 5 | 3250 | 0.200 | 0.024389073 |
| Asthma | 1 | 16 | 5 | 3250 | 0.200 | 0.024389073 |
| Huntington disease | 1 | 16 | 6 | 3250 | 0.167 | 0.029199482 |
| Leishmaniasis | 1 | 16 | 6 | 3250 | 0.167 | 0.029199482 |
| Human cytomegalovirus infection | 1 | 16 | 6 | 3250 | 0.167 | 0.029199482 |
| Long-term potentiation | 1 | 16 | 7 | 3250 | 0.143 | 0.033987647 |
| Spinocerebellar ataxia | 1 | 16 | 7 | 3250 | 0.143 | 0.033987647 |
| Nicotine addiction | 1 | 16 | 7 | 3250 | 0.143 | 0.033987647 |
| Glutamatergic synapse | 1 | 16 | 8 | 3250 | 0.125 | 0.038753665 |
| Cocaine addiction | 1 | 16 | 8 | 3250 | 0.125 | 0.038753665 |
| Necroptosis | 1 | 16 | 9 | 3250 | 0.111 | 0.043497633 |
| Circadian entrainment | 1 | 16 | 9 | 3250 | 0.111 | 0.043497633 |
| GABAergic synapse | 1 | 16 | 9 | 3250 | 0.111 | 0.043497633 |
| Long-term depression | 1 | 16 | 9 | 3250 | 0.111 | 0.043497633 |
| Amphetamine addiction | 1 | 16 | 10 | 3250 | 0.100 | 0.048219644 |
| Alcoholism | 1 | 16 | 10 | 3250 | 0.100 | 0.048219644 |

**Table S6 Metabolic pathway enrichment of differential metabolites between Proximal T and Proximal N**

| **Annotation** | **in set** | **set** | **in background** | **background** | **RichFactor** | **p-value** |
| --- | --- | --- | --- | --- | --- | --- |
| Aminoacyl-tRNA biosynthesis | 4 | 21 | 52 | 3250 | 0.077 | 0.000285357 |
| Central carbon metabolism in cancer | 3 | 21 | 30 | 3250 | 0.100 | 0.000844206 |
| GABAergic synapse | 2 | 21 | 9 | 3250 | 0.222 | 0.001393314 |
| Alcoholism | 2 | 21 | 10 | 3250 | 0.200 | 0.001734863 |
| ABC transporters | 4 | 21 | 93 | 3250 | 0.043 | 0.002588677 |
| Purine metabolism | 4 | 21 | 95 | 3250 | 0.042 | 0.002798826 |
| D-Glutamine and D-glutamate metabolism | 2 | 21 | 13 | 3250 | 0.154 | 0.002972134 |
| Histidine metabolism | 3 | 21 | 47 | 3250 | 0.064 | 0.003141398 |
| Sphingolipid signaling pathway | 2 | 21 | 15 | 3250 | 0.133 | 0.003969898 |
| Proximal tubule bicarbonate reclamation | 2 | 21 | 17 | 3250 | 0.118 | 0.005102079 |
| Parkinson disease | 2 | 21 | 20 | 3250 | 0.100 | 0.007045187 |
| Taurine and hypotaurine metabolism | 2 | 21 | 22 | 3250 | 0.091 | 0.008499114 |
| Biosynthesis of unsaturated fatty acids | 3 | 21 | 69 | 3250 | 0.043 | 0.009263589 |
| Arginine biosynthesis | 2 | 21 | 23 | 3250 | 0.087 | 0.009272445 |
| Alanine, aspartate and glutamate metabolism | 2 | 21 | 28 | 3250 | 0.071 | 0.013587294 |
| Protein digestion and absorption | 2 | 21 | 29 | 3250 | 0.069 | 0.014537245 |
| beta-Alanine metabolism | 2 | 21 | 32 | 3250 | 0.063 | 0.017554373 |
| Glutathione metabolism | 2 | 21 | 38 | 3250 | 0.053 | 0.024309003 |
| Apoptosis | 1 | 21 | 4 | 3250 | 0.250 | 0.02560843 |
| Neuroactive ligand-receptor interaction | 2 | 21 | 40 | 3250 | 0.050 | 0.026763714 |
| Butanoate metabolism | 2 | 21 | 42 | 3250 | 0.048 | 0.029315469 |
| FoxO signaling pathway | 1 | 21 | 5 | 3250 | 0.200 | 0.031912257 |
| Huntington disease | 1 | 21 | 6 | 3250 | 0.167 | 0.038177232 |
| Ascorbate and aldarate metabolism | 2 | 21 | 49 | 3250 | 0.041 | 0.03897296 |
| Long-term potentiation | 1 | 21 | 7 | 3250 | 0.143 | 0.044403581 |
| Spinocerebellar ataxia | 1 | 21 | 7 | 3250 | 0.143 | 0.044403581 |
| Nicotine addiction | 1 | 21 | 7 | 3250 | 0.143 | 0.044403581 |
| Nicotinate and nicotinamide metabolism | 2 | 21 | 55 | 3250 | 0.036 | 0.048088513 |
