# Supplementary figures and images for: Microbiota and metabolites alterations in proximal and distal gastric cancer patients
Source: J Transl Med. 2022 Sep 30;20:439. doi: 10.1186/s12967-022-03650-x (PMC9524040; doi:10.1186/s12967-022-03650-x)

# Phylum

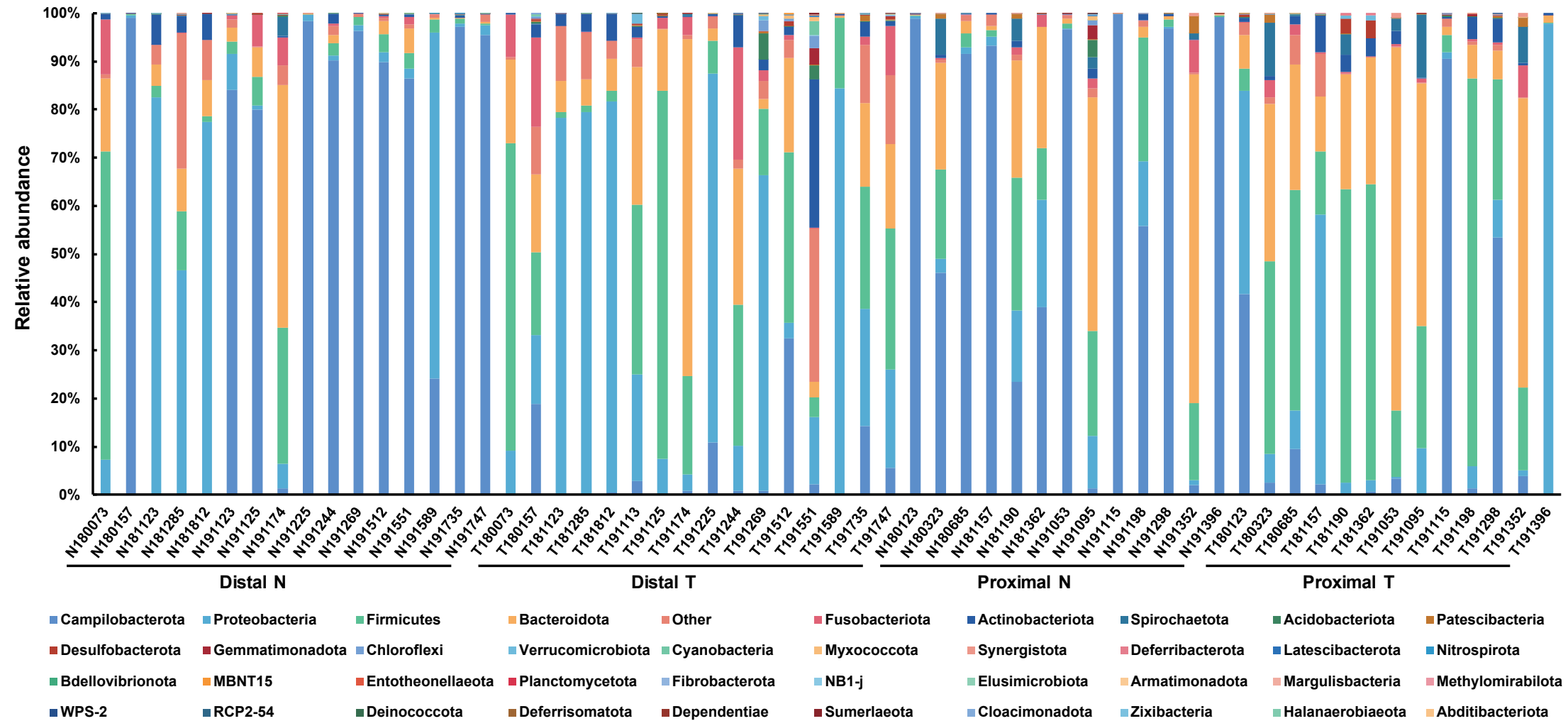

Supplement: Supplementary file 2 — Additional file 2: Figure S2. The microbial relative abundance of proximal, distal tumor tissues and matched non-tumor tissues at the genus level. Proximal T,proximal GC tumor tissues; Proximal N,proximal GC non-tumor tissues; Distal T, distal GC tumor tissues; Distal N, distal GC non-tumor tissues. [file 12967_2022_3650_MOESM2_ESM.pdf]

## Genus

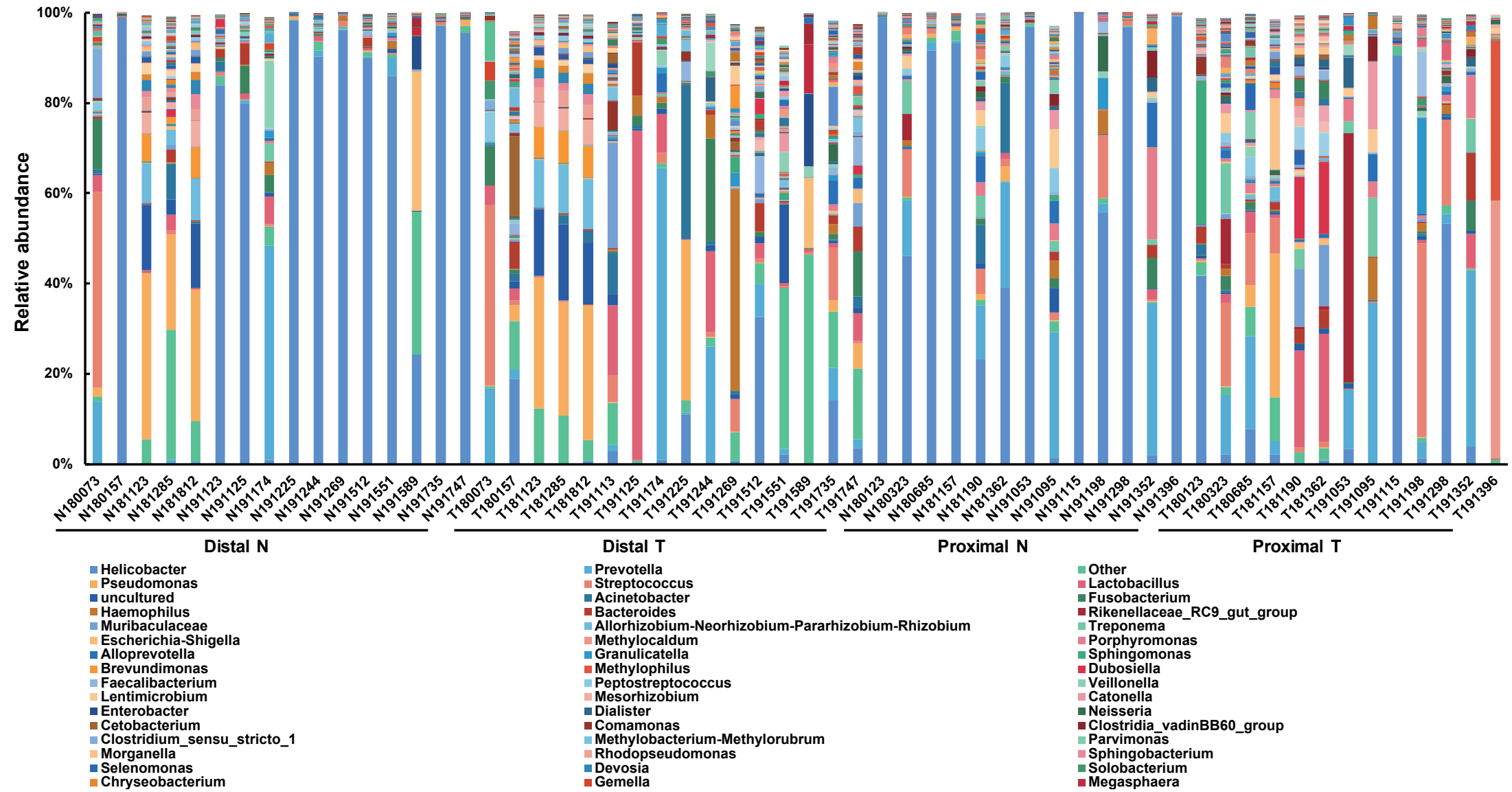

Supplement: Supplementary file 3 — Additional file 3: Figure S2. The microbial relative abundance of proximal, distal tumor tissues and matched non-tumor tissues at the genus level. Proximal T,proximal GC tumor tissues; Proximal N,proximal GC non-tumor tissues; Distal T, distal GC tumor tissues; Distal N, distal GC non-tumor tissues. [file 12967_2022_3650_MOESM3_ESM.pdf]

A)

Distal N Distal T

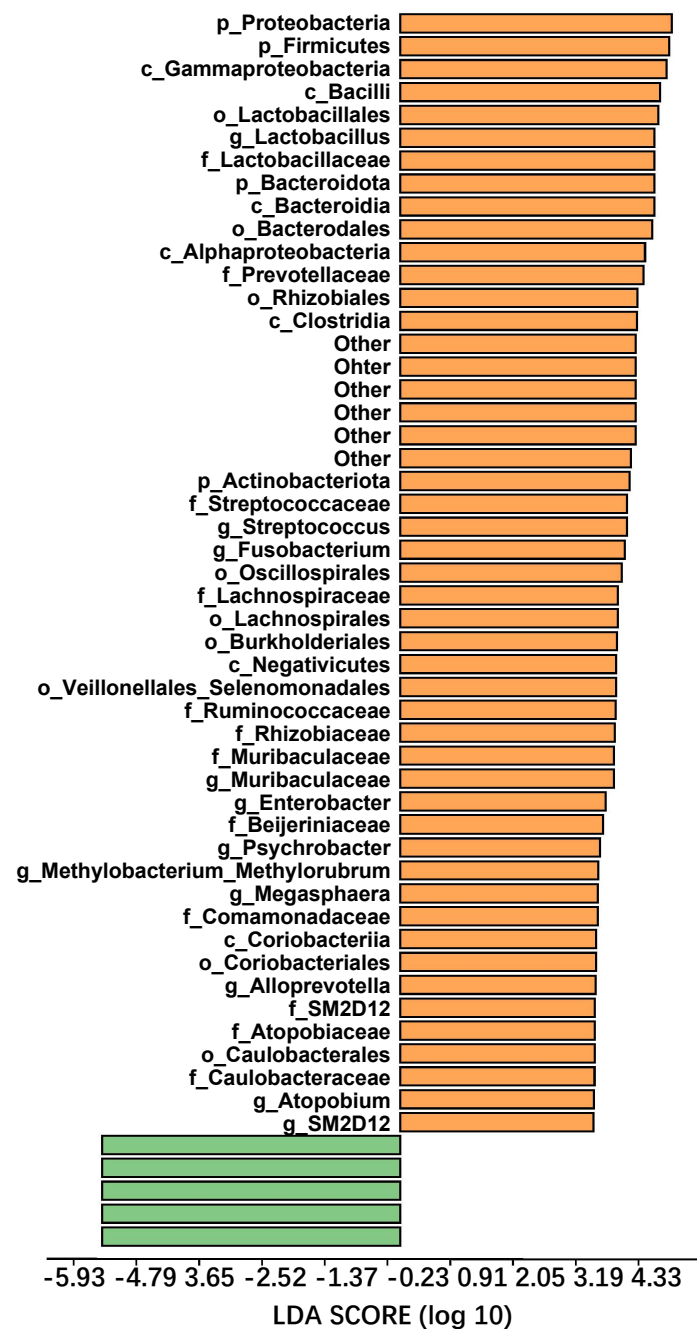

B)

Proximal N Proximal T

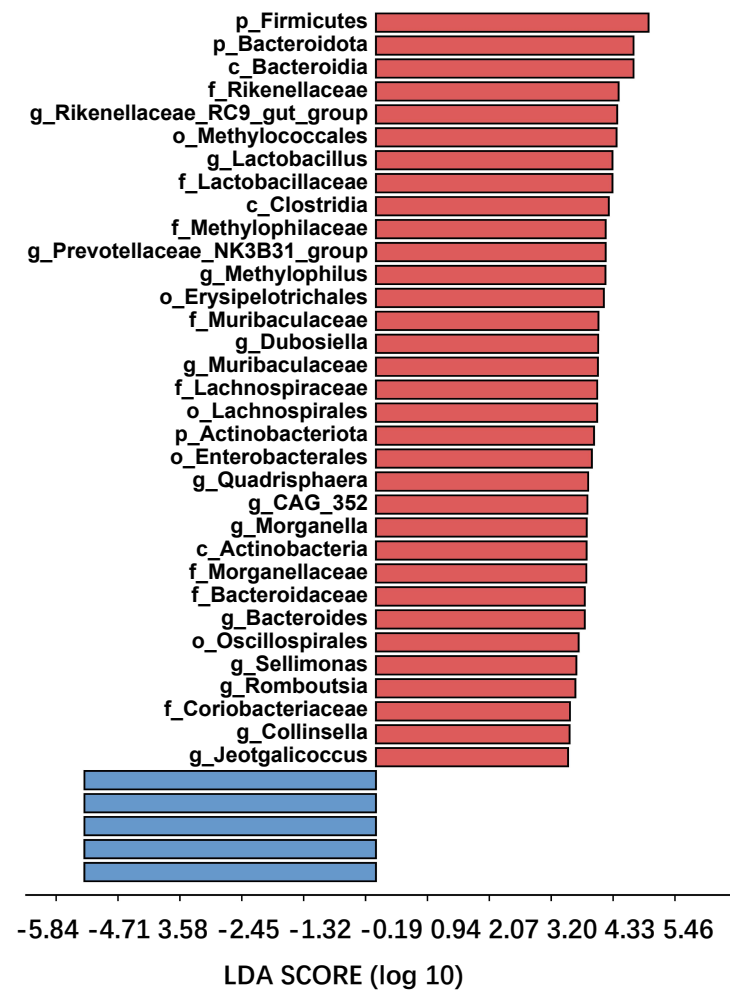

Supplement: Supplementary file 4 — Additional file 4: Figure S3. Differential microbiota of gastric cancer tissues and non-tumor tissues. A Differential taxa from phylum to genus of Distal T and Distal N. B Differential taxa from phylum to genus of Proximal T and Proximal N. [file 12967_2022_3650_MOESM4_ESM.pdf]

A)

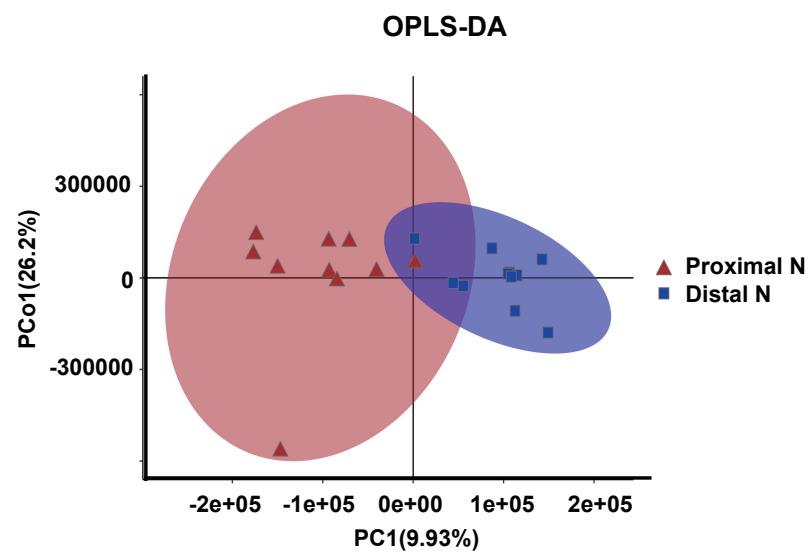

B)

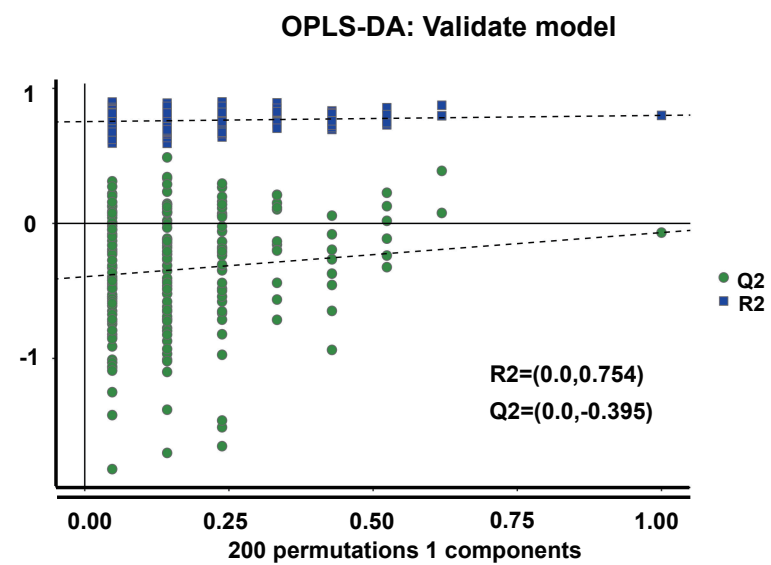

Supplement: Supplementary file 5 — Additional file 5: Figure S4. Metabolite composition and difference between Proximal N and Distal N. A, B OPLS-DA showed that Proximal N and Distal N were not separated into two clusters. Test for OPLS-DA model showed that the OPLS-DA model for this study was valid. [file 12967_2022_3650_MOESM5_ESM.pdf]
